# Chemical Diversity and Anti-Insect Activity Evaluation of Essential Oils Extracted from Five *Artemisia* Species

**DOI:** 10.3390/plants11131627

**Published:** 2022-06-21

**Authors:** Jia-Wei Zhang, Bo-Ya Li, Xin-Xin Lu, Yu Zheng, Dan Wang, Zhe Zhang, Ding Zeng, Shu-Shan Du

**Affiliations:** 1 Beijing Key Laboratory of Traditional Chinese Medicine Protection and Utilization, Faculty of Geographical Science, Beijing Normal University, No.19 Xinjiekouwai Street, Beijing 100875, China; jiawei-zhang@mail.bnu.edu.cn (J.-W.Z.); 17862963570@163.com (X.-X.L.); zhengyu123@mail.bnu.edu.cn (Y.Z.); zhezhang@mail.bnu.edu.cn (Z.Z.); 2 Department of Biomedical Science, Beijing City University, No. 269 North 4th Ring Middle Road, Beijing 100083, China; li_boya@foxmail.com (B.-Y.L.); wangdan@bcu.edu.cn (D.W.); 3 Department of Burns and Plastic Surgery, PLA Rocket Force Characteristic Medical Center, Beijing 100088, China

**Keywords:** *Artemisia*, repellent activity, contact toxicity, *Liposcelis bostrychophila*, *Tribolium castaneum*

## Abstract

As a source of aromatic plants, the genus *Artemisia* has long been considered to have the potential to develop plant pesticides. In this study, components of essential oils from *A. dalai-lamae*, *A. tangutica*, *A. sieversiana*, *A. tanacetifolia* and *A. ordosica* were identified by GC-MS. A total of 56 constituents were analysed, and each species consisted of 9 to 24 constituents. Principle component analysis (PCA) revealed that *A. dalai-lamae*, *A. tangutica* and *A. tanacetifolia* are characterised by monoterpene hydrocarbons and oxygenated monoterpenes. Hierarchical cluster analysis (HCA) showed the most remarkable similarity between *A. sieversiana* and *A. ordosica*, but the similarity was still lower than 50%. Contact toxicity and repellency of essential oils were evaluated by bioassays; *A. ordosica* oil exhibited the most substantial contact toxicity (LD_50_ = 52.11 μg/cm^2^) against *Liposcelis bostrychophila*, while *A. tangutica* oil showed the most potent contact toxicity (LD_50_ = 17.42 μg/adult) against *Tribolium castaneum*. Except for *A. dalai-lamae*, the other four species showed the same level (*p* > 0.05) of repellent activity as the positive control against both pests at high concentrations. The results indicated that these five *Artemisia* species had high chemical diversity and great potential to be developed into more effective and environmentally friendly anti-insect agents.

## 1. Introduction

Insecticides are an effective method of controlling storage pests, of which synthetic insecticides have been demonstrated to impact environmental protection and health safety negatively [1,2]. This phenomenon promoted the growing exploration of botanical pesticides. Plant secondary metabolites are important sources of botanical insecticides and have proven insecticidal [3,4] and repellent [5,6] effects in practice. Increasing studies have shown that essential oils mainly extracted from aromatic plants have great potential against arthropod species [7]. The genus *Artemisia*, one of the largest genera of the family Compositae, consists of nearly 500 species worldwide, and about 190 species are found in China. High concentrations and significant intraspecific variations of volatile terpenes in essential oil generated the strong and diverse odour of the *Artemisia* genus [8]. The compositional diversity of essential oils of several *Artemisia* species has been reported. *A. dracunculus* was rich in (*Z*)-anethole (81.0%) [9], *A. scoparia* was rich in α-thujone (81.7%) [10], while 67% of camphor was the main component of *A. fragans* [11].

The positive repellent and insecticidal activities of essential oils derived from *Artemisia* species have been in the limelight owing to the abundant volatile components [8,12]. Essential oils extracted from three *Artemisia* species, including *A. absinthium*, *A. spicigera* and *A. santonicum*, were toxic to *Sitophilus granaries* [13]. Oils from cultivated *A. absinthium* had better repellent activity against *Trypanosoma cruzi* and *Leishmania infantum* than the commercial ones [14]. *A. capillaris* and *A. mongolica* essential oils showed significant toxicity against *Sitophilus zeamais* [15]. 

Little research was conducted on *A. dalai-lamae* and *A. tangutica*, the endemic species in China. Terpenoids, lignans, flavonoids, and various compounds were isolated from *A. sieversiana*. Some of these components showed several biological activities such as anti-tumour, anti-inflammatory, anti-allergic, anti-hypertensive, and anti-hyperglycemic activities [16]. The methanolic extract of the leaves of *A. tanacetifolia* afforded several kinds of coumarins [17], and 5-*O*-caffeoylquinic acid was detected from the aerial parts of flowering *A. tanacetifolia* [18]. Essential oil from *A. ordosica* has the effectiveness of anti-inflammatory, haemostasis, treating rheumatoid arthritis, parotiditis, abdominal distension, intestinal obstruction and ischuria [19]. Although numerous studies have shown that *Artemisia* species has various pharmacological effects, still little is known concerning the insecticidal activity of these five *Artemisia* species.

In this study, we reported the essential oils from five species in the genera *Artemisia* (*A. dalai-lamae*, *A. tangutica*, *A. sieversiana*, *A. tanacetifolia* and *A. ordosica*) and provided a comparative investigation of these five species’ chemical composition, repellent, and contact potential for *Liposcelis bostrychophila* Badonnel and *Tribolium castaneum* Herbst adults.

## 2. Results

### 2.1. Chemical Composition of the Essential Oils

Essential oils of the five plants aerial parts were obtained by hydrodistillation and analysed by GC-MS. The major chemical compounds are presented in Table 1. The data of *A. ordosica* were drawn from Zhang et al. (2017) [20]. The yields of five *Artemisia* species essential oils ranged from 0.02% to 0.53% (*v*/*w*%, Table 2). The chemical composition of these oils is different. *A. tanacetifolia* essential oil contains substantial amounts of 3-carene (45.98%) and *β*-pinene (15.13%), which were absent in other samples. The major components of *A. sieversiana* are neryl propanoate (22.88%), *β*-nerol (11.01%) and *β*-cubebene (7.50%), whereas cineol (32.62%), 3,7-dimethyl-1,5,7-octatriene-3-ol (15.85%), and santolina triene (14.45%) are the major compounds of *A. dalai-lamae*. Unlike *A. dalai-lamae*, *A. ordosica* yielded oil which is rich in caryophyllene (17.81%), *β*-bisabolene (12.11%), spathulenol (10.56%) and *β*-caryophyllene oxide (8.67%). In addition, the five essential oils also have similar sets of main components: caryophyllene (0.91–17.81%), camphor (1.32–51.07%), linalool (0.56–1.34%), *α*-terpineol (0.92–3.69%), 4-terpineol (1.12–11.97%) and nerolidol (0.24–1.47%) existing in at least three oils.

**Figure 1:**
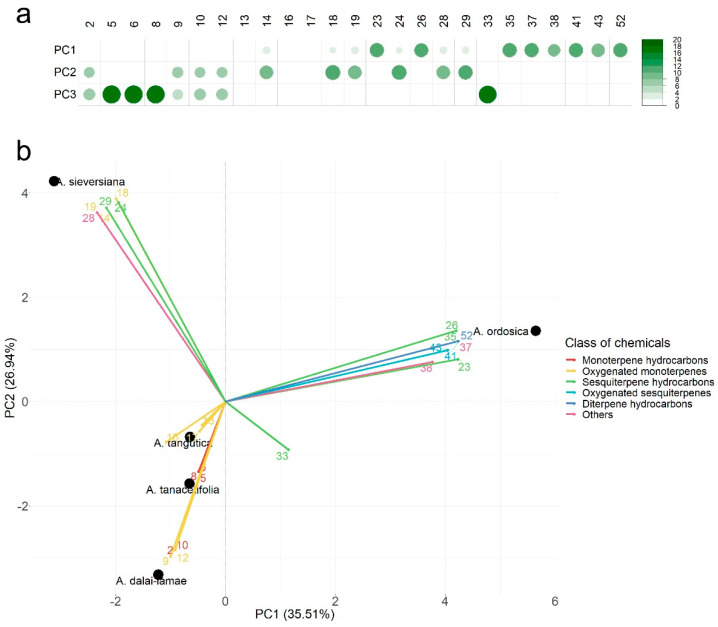
Principal component analysis (PCA) of five *Artemisia* species according to the major essential oil compositions (concentration > 3%). (**a**) the contribution (%) of chemical constituents to the first two principal components; (**b**) bi-plot of PCA. The serial numbers of components are consistent with those in Table 1.

**Figure 2:**
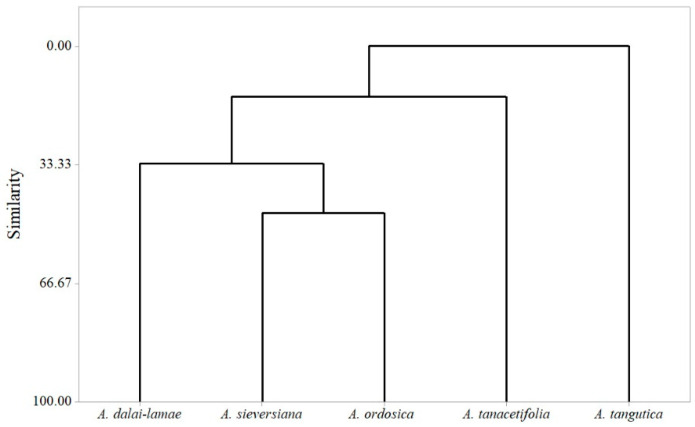
Dendrogram analysis based on essential oil components of five *Artemisia* species.

Twenty-five compounds with a concentration > 3% (for these components were rich enough for describe the characteristics of essential oils) were selected to perform PCA. PCA was used to determine the impacts and differences of the most important compounds. The contributions to the first two components of each chemical are shown in Figure 1a. *β*-Elemene (23), caryophyllene (26), *β*-bisabolene (35), myristicine (37), *β*-caryophyllene oxide (41) and (*E*)-phytol (52) gave the contribution of over 10% to PC1; *α*-terpineol (18) *β*-cubebene (24) and elixene (29) gave over 10% of contribution to PC2; over 15% of contribution was contributed by *β*-pinene (5), sylvestrene (8) and germacrene D (33). The bi-plot of PCA is shown in Figure 1b. PC1 and PC2 described 62.45% of the total variances. PC1 had the highest positive correlation with 3-carene (3), and the highest negative correlation with camphor (14). PC2 was represented mainly by cineol (9) in the positive score. *A. dalai-lamae*, *A. tangutica* and *A. tanacetifolia* were characterised by monoterpene hydrocarbons and oxygenated monoterpenes. *A. sieversiana* was characterised by oxygenated monoterpenes, sesquiterpene hydrocarbons and neryl propanoate (28). Moreover, sesquiterpene hydrocarbons, oxygenated sesquiterpenes, and a diterpene, (*E*)-phytone, etc., could describe the chemical character of *A. ordosica*. A dendrogram of HCA revealed the similarity of these *Artemisia* species (Figure 2). *A. sieversiana* and *A. ordosica* had the greatest similarity, but the similarity rate was lower than 50%. *A. tangutica* was spread from the other four species with a minimum likeness. 

### 2.2. Repellent Activity

**Figure 3:**
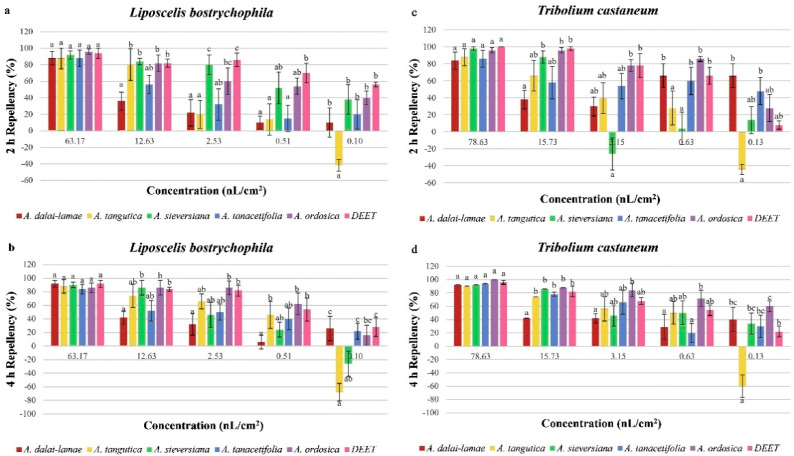
Percentage repellency (PR) value of five *Artemisia* species essential oil against *L. bostrychophila* (**a**,**b**) and *T. castaneum* (**c**,**d**) adults at 2 h (**a**,**c**) and 4 h (**b**,**d**) after exposure. Means in the same column followed by the same letters do not differ significantly (*p* > 0.05) in ANOVA and Tukey’s tests.

The repellent rates at 2 and 4 h after exposure to essential oils derived from five *Artemisia* species against *L. bostrychophila* and *T. castaneum* are shown in Figure 3, respectively. The repellent effect of essential oils on both two pests showed various levels. When compared with the positive control, DEET, at both 2 and 4 h after exposure, all five oils possessed the same level of repellent activity (*p* > 0.05) at a testing concentration of 63.17 nL/cm^2^ for L. bostrychophila and 78.63 nL/cm^2^ for *T. castaneum*. The essential oil of *A. tangutica*, *A. sieversiana*, *A. tanacetifolia* and *A. ordosica* also showed comparable repellent levels with the positive control (*p* > 0.05) at the concentration of 12.63 nL/cm^2^ against *L. bostrychophila* and 15.73 nL/cm^2^ for *T. castaneum* adults. It is worth noting that *A. tangutica* and *A. sieversiana* were found to have attraction effects against these two insects at the lowest concentration. Among the oils, *A. ordosica* showed outstanding repellent activity and possessed the same (*p* > 0.05) with DEET at all five concentrations on both insects.

### 2.3. Contact Toxicity

The contact toxicities of essential oils from five *Artemisia* species against *L. bostrychophila* and *T. castaneum* adults are listed in Table 3 and Table 4. Except for *A. tanacetifolia*, others all exhibited contact effects against *L. bostrychophila*. Among them, the *A. ordosica* essential oil possessed the most potent contact toxicity (LD_50_ = 52.11 μg/cm^2^), about three times less than the positive control, pyrethrins. Compared with *A. ordosica*, *A. tangutica* essential oil showed slightly weaker activity with an LD_50_ value of 70.48 μg/cm^2^. In addition, both *A. sieversiana* and *A. dalai-lamae* essential oils exhibited moderate toxicities with LD_50_ values of 195.51 and 115.94 μg/cm^2^, respectively, while for the insect of *T. castaneum* adults, *A. tangutica* possessed the most substantial toxicity with an LD_50_ of 17.42 μg/adult, followed by *A. ordosica*, *A. dalai-lamae* and *A. tanacetifolia*, with LD_50_ values of 21.68, 25.70 and 41.90 μg/adult, respectively.

## 3. Discussion

The chemical composition of *Artemisia* species oils has high diversity. The oil has different protective effects due to various compositions, such as antibacterial activity, insecticidal effect, antiviral and repellent actions. 

Only essential oil components from *A. sieversiana* and *A. ordosica* have been reported before. Reports of the chemical constitutions of essential oils from these two kinds of *Artemisia* species indicated the high diversity caused by geographic locations (which may affect precipitation, temperature, edatope, etc.). The compositions of essential oils of *A. sieversiana* gathered from different regions of China in the flowering stage had a high level of variability. When sampling from Beijing, the essential oil contained eucalyptol (9.2%), geranyl butyrate (9.1%), camphor (7.9%), borneol (7.9%) and germacrene D (5.5%) [27], whereas essential oils of *A. sieversiana* gathered from Tibet mainly consisted of *α*-bisabolol (34.47%), chamazulene (23.00%) and *α*-phellandrene (5.22%) [28]. In Ningxia, the oil was mainly chamazulene (29.61%), camphor (4.80%) and eucalyptol (4.32%) [29].

The essential oil extracted from *A. ordosica* collected from the southwestern boundary of Tengger Desert was mainly magnol (22.60%), *trans*-*β*-ocimene (11.60%), and acenaphthylene (11.00%) [30]. 2,5-Etheno[4.2.2]propella-3,7,9-triene (24.81%), *trans*-nerolidol (10.39%) and *α*-longipinene (8.82%) were the predominant compounds of the sample collected from the southeastern boundary of the Hopq desert [31]. In the Mu Us desert, *β*-pinene (11.17%), limonene (11.41%) and capillene (9.46%) were the major components [32].

In our tests, the main compounds of tested oils also were found in other *Artemisia* species. For example, camphor, linalool and 4-terpineol were the major compositions in *A. haussknechtii* [33], and spathulenol, *β*-elemene, germacrene D were found in *A. campestris* [34]. Caryophyllene was the main compound in *A. lavandulaefolia* and *A. rubripes* oils [35,36]. 

It has also been reported that changes in the composition of volatile oils can also occur during plant growth stages. For instance, monoterpenoids, the major composing components (69.5–77.7%) of *Ocimum americanum* oil, were found to be maximal (77.7%) in the vegetative growth stage followed in the seed setting period (76.8%) and full flowering stage (74.2%), with the minimum at the half-flowering stage (69.5%) [37]. 

Recently, thousands of plants have been deemed as potential sources of repellents. The repellent properties of essential oils from the genus *Artemisia* were also well documented. In our previous research, five *Artemisia* species, including *A. anethoides*, *A. giraldii*, *A. roxburghiana* and *A. rubripes* were evaluated for their repellent activities on *T. castaneum* [38]. The result indicated that the five essential oils were effective in repelling *T. castaneum,* and the sequence of their activity was *A. rubripes* > *A. anethoides* > *A. roxburghiana* = *A. sacrorum* = DEET (the positive control) > *A. giraldii* (*p* > 0.05). In another previous research, polyacetylenes were isolated from the essential oil of *A. ordosica* aerial parts. Although with low relative content, the three tested polyacetylenes (capillene, capillin and *cis*-dehydromatricaria ester) were proved to possess fair repellent and fumigant activities against *T. castaneum* adults. Additionally, *A. lavandulaefolia* essential oil and its six constituents were tested on *Lasioderma serricorne* [39]. At 2 h after exposure, the same level of repellency (*p* > 0.05) was observed at doses from 0.63 to 78.63 nL/cm^2^. In other reports, *A. vulgaris* essential oil presented high repellent activity against *T. castaneum* [40]. The essential oil extracted from *A. scoparia* had more marked repellent activity on *Sitophilus oryzae* and *T. castaneum* than *Callosobruchus maculatus*, but as a whole, the oil strongly repelled each species of tested pests [41]. Moreover, in our tests, the main constituents of the five *Artemisia* species were proved to have a repellent effect. For example, 3-carene had over 85% of PR values against *L. bostrychophila* and *T. castaneum* after 2 h exposure [22]. Caryophyllene had the PR values of 82% and 98% against *T. castaneum* after 2 h and 4 h exposure, respectively [22]. These major components were also confirmed to be toxic to other insects, such as *Aedes aegypti*, *Semanstus japonicus* and *Lasioderma serricorne* [42,43,44]. Therefore, this study and the previous reports proved that essential oils from the genus *Artemisia* have great potential to be developed as good repellent agents against storage insects. 

The different toxicity effects could be found based on the LD_50_ values of five *Artemisia* species oils obtained in this study. The essential oils from *A. ordosica* and *A. tangutica* exhibited stronger contact toxicity than others against *L. bostrychophila* and *T. castaneum*. The essential oil of *A. sieversiana* possessed weak toxicity against *L. bostrychophila* with a LD_50_ value of 195.51 μg/cm^2^, and no insecticide effect was observed in tested concentrations to *T. castaneum*. In the previous report, *A. sieversiana* also possessed weak contact toxicity against *Sitophilus zeamais* adults with an LD_50_ value of 112.7 mg/adult [27]. No significant correlation was observed when combined with the LD_50_ values of principal components and essential oils. This is considered to be related to the content of components and the synergistic or antagonistic effect between compounds. Pavela et al. (2010) estimated the fumigant toxicity against *Spodoptera littoralis* larvae of 15 pairs of binary mixtures [45]. It showed that nine mixtures had a synergistic effect, five mixtures had an additive effect, and one mixture had an antagonistic effect. It was also identified that 138 synergistic/antagonistic effects were detected among 39 compounds in binary mixtures via topical application against *Trichoplusia ni* [46]. So it may need further study to reveal the relationship between the insecticidal activities of different compounds.

The mechanism of bio-action of essential oils was recorded. For contact toxicity, substances could permeate through the skin of insects to act on the insects. Some of the substances could cause neurological disorders, for example, the inhibition of the activity of acetylcholinesterase (AChE), which is an important enzyme to regulate synaptic transmission [47]. The inhibition of AChE could lead to hyperexcitation, causing locomotor behaviour alternation. High concentrations or combined use of substances may generate the knockdown effect, reflecting acute toxicity [48]. These phenomena make us care about the safety of applying essential oils and plant-derived substances. Although the potential toxicity to mammals or cells of these five *Artemisia* species has not been studied yet, the safety evaluation of some other species in *Artemisia* has been recorded. The water extracts of *A. dracunculus* showed a maximum tolerated dose to rodents at over 200 mL of extract (1:10)/kg bw [49]. Essential oil from *A. nilagirica* had a LD_50_ value of 7528.10 µL/kg for male mice [50]. The essential oil of *A. herba-alba* showed no significant cytotoxicity in macrophages at the concentrations of 1.25 µL/mL and no significant cytotoxicity to microglial cells and keratinocytes at concentrations up to 0.32 µL/mL [51]. Moreover, the safety of the major constituents, 1,8-cineole, camphor, 3-carene and caryophyllene, was evaluated. After acute oral administration of 1,8-cineole, the LD_50_ value was 3849 mg/kg, while no significant changes in body weight and relative organ weight were observed in the subacute toxicity study [52]. The natural form of camphor was not toxic at 100 mg·kg·b.w.^−1^ to mice [53]. For 3-carene, the exposure by inhalation at 0.0014 mg/day was believed to be safe, and it could cause eye irritation at about 2.39 mg/mL air [54,55]. Caryophyllene was not considered as a skin sensitiser, and the TTC (threshold of toxicological concern) of inhalation exposure was 0.012 mg/day, which is 117 times lower than the Cramer Class I TTC [56]. However, essential oils from some Artemisia species were confirm to have toxicity to human. For example, volatile oil extracted from A. argyi could cause hepatocellular harm to cause liver injury [57]. Although there was no direct evidence that these five essential oils are safe enough for use, combined with the toxicity of other essential oils from *Artemisia* and the major constituents, it can be assumed that these essential oils are at lower concentrations of safe use as insecticides.

## 4. Materials and Methods

### 4.1. Plant Material

Plant materials were included the aerial parts of *A. dalai-lamae*, *A. tangutica*, *A. sieversiana*, *A. tanacetifolia* and *A. ordosica*. They were collected in the Gansu, Xinjiang and Inner Mongolia provinces, China, respectively, and voucher specimens were deposited at the herbarium of Faculty of Geographical Science, Beijing Normal University. The species of plants were identified by Dr. Liu, Q.-R. (College of Life Sciences, Beijing Normal University, Beijing, China). Table 2 summarises the collecting information of these samples. 

### 4.2. Extraction and GC-MS Analysis of Essential Oils

The plant materials were dried in the shade and coarsely ground. Then, the grounded materials were subjected to 6 h of hydrodistillation using a modified Clevenger type apparatus, and the cooled essential oils were dehydrated by anhydrous sodium sulphate. The final oils were stored in sealed containers in the refrigerator at 4 °C.

Gas chromatographic-mass spectrometry (GC-MS) analysis was performed with an Agilent 6890N gas chromatograph (Agilent Technologies, DE, USA) equipped with a flame ionisation detector (FID). A HP-5MS (30 m × 0.25 mm × 0.25 μm) capillary column was used to distinguish the compounds. A 1.0 mL/min flow rate helium was used as a carrier gas. Then, 1 μL of 1% essential oil–*n*-hexane solution was injected, and the injector temperature was 250 °C. The oven temperature was programmed as follows as 50 °C for 2 min, increased at 2 °C/min to 150 °C for 2 min, then increased at 10 °C/min to 250 °C for 5 min.

Constituents were identified by comparing their retention indices (RI) with those reported in the literature, and by matching their mass spectra with those stored in NIST 05, Wiley 275 libraries or literature [58]. The GC-FID peak area (%) was used to obtain the relative percentages of each individual component of the essential oils.

### 4.3. Insects

A 10:1:1(*w*/*w*/*w*) mixture of flour, milk powder and yeast was used to rear *L. bostrychophila*, whether *T. castaneum* was fed with wheat flour mixed with yeast (10:1, *w*/*w*). The colonies were maintained in the dark incubators at 28–29 °C and 70–80% RH. The mixed-sex adults used in repellent and contact assays were about 7 ± 2 days old. The edges of containers and the Petri dishes for *L. bostrychophila* were smeared with polytetrafluoroethylene to ensure escape-proofing.

### 4.4. Repellent Activity

The repellence assay was performed using the area preference method [59]. Five doses of 63.17, 12.63, 2.53, 0.51 and 0.10 nL/cm^2^ were made by serial dilution in *n*-hexane for the repellent assay applied against *L. bostrychophila* adults. Filter papers with a 5.5 cm diameter were cut in half. Each tested solution (150 μL) was applied to half-cut filter paper as the experimental group, and *n*-hexane (150 μL) was used in another half as the control group. The treated filter papers were air-dried to evaporate the solvent completely. Then, both semi-circular filter papers were attached to their opposite sides and placed in Petri dishes (Φ = 5.5 cm). Meanwhile, for *T. castaneum*, the filter papers and Petri dishes were prepared at 9 cm in diameter. The five tested concentrations were 78.63, 15.73, 3.15, 0.63 and 0.13 nL/cm^2^, and 500 μL of testing solution or *n*-hexane were treated on each semi-circular filter paper. Twenty insects were released at the centre of the Petri dishes and covered with lids for all tests. The dishes were then placed in the dark incubators in the same condition as raring. Five replications were used for each concentration. The positive control was conducted by DEET (*N*, *N*-diethyl-3-methylbenzamide, Dr. Ehrenstorfer, Germany). The numbers of insects present on different sides of the paper were recorded after 2 and 4 h.

### 4.5. Contact Toxicity

The contact toxicity of the essential oils was conducted as follows. The appropriate testing concentrations were determined for all bioassays based on range-finding studies. Then, the desired quantity of each sample was dissolved in *n*-hexane to obtain a series of concentrations as the testing solution. When it came to *L. bostrychophila* adults, 300 μL of the solutions of the essential oils were applied to a round filter paper of 5.5 cm in diameter. Then the treated filter paper was attached to the bottom of Petri dishes of the same size as the filter papers. Ten insects in each treatment were put in the Petri dishes. All the Petri dishes were covered by lids and kept in the incubator. For the bioassays with *T. castaneum* adults, 0.5 μL of solutions were applied to the insects’ dorsal thorax. Ten treated insects with the same solution were transferred into one vial, and reared in the incubator. 

After 24 h, the number of deaths was checked and recorded. The *n*-hexane was used as the negative control, and pyrethrin (pyrethrin I and II, 37%) was used as the positive control. The experiments were replicated five times.

### 4.6. Statistic Analysis

Principle component analysis (PCA) was used to explain the dissimilarities between samples. Hierarchical cluster analysis (HCA) was used to evaluate the similarity of the samples based on the type and quantity of compounds from essential oils. PCA was conducted by R Studio (version 4.1.3) with FactoMineR [60] and factoextra [61] packages. HCA was performed with Minitab 17 by using the complete linkage and Euclidean distance measure methods. A dendrogram was constructed to express the result.

The percentage repellency (PR) was calculated to measure the repellent activity of essential oils, which was computed by the foluma as below [59]:

$$\mathrm{PR}\left(\%\right)=\left[\left(\mathrm{Nc}-\mathrm{Nt}\right)/\left(\mathrm{Nc}+\mathrm{Nt}\right)\right]\times 100$$

Nc and Nt are the numbers of insects in the negative control and treated half, respectively. Then the analysis of variance (one-way ANOVA) and Tukey’s test were conducted using SPSS 20.0. 

For the contact toxicity test, LD_50_ was calculated by Probit analysis using SPSS 20.0. The 95% FL (fiducial interval), Slope ± SE, *p*-value and χ^2^ were also recorded.

## 5. Conclusions

The chemical composition, repellent activity and contact toxicity of five *Artemisia* species (*A. dalai-lamae*, *A. tangutica*, *A. sieversiana*, *A. tanacetifolia* and *A. ordosica*) essential oils extracted by hydrodistillation were compared by PCA and HCA. All the oils showed obvious repellent activity against both insects in repellent tests. As for contact toxicity, the obtained values of LD_50_ demonstrated that several of the evaluated essential oils possessed toxic effects on *L. bostrychophila* and *T. castaneum* adults. Among these oils, *A. ordosica* essential oil possessed the most potent contact toxicity (LD_50_ = 52.11 μg/cm^2^) against *L. bostrychophila*, and *A. tangutica* essential oil exhibited the most substantial toxicity (LD_50_ = 17.42 μg/adult) against *T. castaneum*. Hence, the results suggest that the above five *Artemisia* species have the potential to be further exploited as repellent and insecticide agents against storage pests.

## Figures and Tables

**Table 1:** Chemical composition of essential oils extracted from the five *Artemisia* species.

No.	RI ^1^	Compound	Relative Content (%) ^2^				
			AD	AG	AS	AC	AO ^3^
1	800	Octane	1.24	-	-	-	-
2	908	Santolina triene	14.45	-	-	-	-
3	925	*α*-Thujene	-	-	-	2.53	-
4	966	*β*-Thujene	-	-	-	2.63	-
5	980	*β*-Pinene	-	-	-	15.13	-
6	1010	3-Carene	-	-	-	45.98	-
7	1021	*α*-Cymene	1.51	-	-	-	-
8	1027	Sylvestrene	-	-	-	5.92	-
9	1046	1,8-Cineole	32.62	-	0.36	2.56	-
10	1074	3,5-Dimethylethylbenzene	7.48	-	-	-	-
11	1106	Linalool	-	-	1.34	1.27	0.56
12	1108	3,7-Dimethyl-1,5,7-octatriene-3-ol	15.85	-	-	-	-
13	1145	Camphor	-	51.07	1.32	-	1.38
14	1160	Borneol	-	-	6.97	-	-
15	1164	Pinocarvone	0.46	-	-	-	-
16	1175	4-Terpineol	2.02	11.97	1.57	1.12	-
17	1182	Isocitral	-	9.20	-	-	-
18	1190	*α*-Terpineol	-	1.47	3.69	1.27	0.92
19	1232	*β*-Nerol	-	-	11.01	-	-
20	1250	*γ*-Pironene	-	-	-	-	2.41
21	1267	Geraniol	-	-	1.44	0.31	-
22	1372	Copaene	-	-	-	-	1.76
23	1388	*β*-Elemene	-	-	-	1.54	5.56
24	1390	*β*-Cubebene	-	-	7.50	-	0.76
25	1396	3-Methyl-2-pent-2-enyl-cyclopent-2-enone	-	-	1.09	-	-
26	1417	Caryophyllene	0.91	3.76	2.02	0.98	17.81
27	1425	1-Methyl-4-(1-methylethylidene)-2-(1-methylvinyl)-1-vinylcyclohexane	-	-	-	1.29	-
28	1430	Neryl propanoate	-	-	22.88	-	-
29	1432	Elixene	-	-	4.21	-	0.19
30	1441	*β*-Farnesene	-	-	2.73	-	1.23
31	1464	*β*-Humulene	-	-	-	-	1.74
32	1465	*γ*-Muurolene	-	-	-	-	1.33
33	1480	Germacrene D	-	-	-	8.79	3.36
34	1489	Viridiflorene	1.14	-	-	-	-
35	1504	*β*-Bisabolene	-	-	-	-	12.11
36	1508	Himbaccol	-	-	1.21	-	-
37	1513	Myristicine	-	-	-	-	3.19
38	1517	Capillene	-	2.57	-	-	4.04
39	1523	*δ*-Cadinene		-	1.50	-	2.64
40	1557	Germacrene B	0.78	-	3.00	-	-
41	1566	*β*-Caryophyllene oxide	-	-	-	-	8.67
42	1576	Nerolidol	-	-	0.49	0.24	1.47
43	1583	Spathulenol	2.19	-	1.04	-	10.56
44	1606	Humulene oxide II	-	1.99	-	-	-
45	1639	*τ*-Cadinol	-	-	1.19	-	-
46	1650	*α*-Cadinol	-	-	-	0.43	1.70
47	1654	Bisabolol oxide B	-	2.23	-	-	-
48	1678	Dillapiol	-	-	-	-	1.18
49	1688	8-Cedren-13-ol	-	-	1.00	-	-
50	1734	1,4-Dimethyl-7-ethylazulene	-	-	2.76	-	-
51	1846	Phytone	-	-	-	-	2.79
52	2119	(*E*)-Phytol	-	-	-	-	5.64
53	2632	Tetracosanal	-	2.18	-	-	-

^1^ RI, retention index of the chromatography determined on a HP-5MS column using the homologous series of 𝑛-hydrocarbons as reference; ^2^ AD, *A. dalai-lamae*; AG, *A. tangutica*; AS, *A. sieversiana*; AC, *A. tanacetifolia*; AO, *A. ordosica*; ^3^ Data from Zhang et al. [20].

**Table 2:** Collecting information of the five *Artemisia* species.

Species	Date	Province	District	Geographic Coordinate	Life Form	Sample Mass (kg)	Yield (*v*/*w*%)
*A. dalai-lamae*	October 2016	Gansu	Lanzhou	103°45′ E, 36°01′ N	Subshrub	2.70	0.35
*A. tangutica*	October 2016	Gansu	Lanzhou	103°45′ E, 36°01′ N	Perennial herb	2.50	0.29
*A. sieversiana*	July 2017	Hebei	Bashang	117°51′ E, 40°57′ N	Annual or biennial herb	6.30	0.06
*A. tanacetifolia*	July 2017	Hebei	Bashang	117°51′ E, 40°57′ N	Perennial herb	3.10	0.20
*A. ordosica* ^1^	October 2015	Inner Mongolia	Kubuqi Desert	109°44′ E, 40°17′ N	Shrub	3.00	0.39

^1^ Data from Zhang et al. [20].

**Table 3:** Contact toxicity of the essential oils from five *Artemisia* species and major compounds against *L. bostrychophila* adults.

Samples	LD_50_ (μg/cm^2^)	FL (μg/cm^2^)	Slope ± SE	*p*-Value	χ^2^
*A. dalai-lamae*	115.94	104.58–129.58	6.42 ± 0.95	0.193	13.58
*A. tangutica*	70.48	68.20–73.89	12.46 ± 1.45	0.374	19.30
*A. sieversiana*	195.51	177.33–215.97	6.87 ± 0.94	0.851	7.89
*A. tanacetifolia*	less than 50% morality at concentration of 50%				
*A. ordosica*	52.11	51.55–53.87	4.88 ± 0.50	0.664	19.62
1,8-Cineole ^1^	1048.74	1021.95–1096.85	9.50 ± 0.91	-	11.76
Camphor ^1^	207.26	199.78–214.99	13.81 ± 1.47	-	15.87
3-Carene ^2^	223.62	205.65–243.00	5.92 ± 0.62	-	8.52
Caryophyllene ^2^	52.52	43.52–60.83	2.77 ± 0.39	-	9.62
Pyrethrins ^3^	18.72	17.60–19.92	2.98 ± 0.40	0.99	10.56

^1^ Data from Liu et al. [21]; ^2^ Data from Cao et al. [22]; ^3^ Data from Liu et al. [23].

**Table 4:** Contact toxicities of the essential oils from five *Artemisia* species and major compounds against *T. castaneum* adults.

Samples	LD_50_ (μg/adult)	FL (μg/cm^2^)	Slope ± SE	*p*-Value	χ^2^
*A. dalai-lamae*	25.70	23.09–28.49	4.19 ± 0.45	0.681	19.34
*A. tangutica*	17.42	15.17–19.98	3.38 ± 0.45	0.553	16.57
*A. sieversiana*	less than 50% morality at concentration of 50%				
*A. tanacetifolia*	41.90	37.08–47.57	3.88 ± 0.45	0.995	6.17
*A. ordosica*	21.68	19.86–23.59	5.41 ± 5.06	0.944	13.34
1,8-Cineole ^1^	18.83	17.13–20.69	4.86 ± 0.50	-	16.56
Camphor ^2^	less than 50% morality at concentration of 50%				
3-Carene ^3^	63.43	57.16–70.75	4.11 ± 0.45	-	11.67
Caryophyllene ^3^	25.86	22.61–30.24	2.97 ± 0.39	-	13.13
Pyrethrins ^4^	0.26	0.22–0.30	3.34 ± 0.32	0.95	13.11

^1^ Data from Wang et al. [24]; ^2^ Data from Guo et al. [25]; ^3^ Data from Cao et al. [22]; ^4^ Data from Guo et al. [26].

## Data Availability

Not applicable.
